# New insights in application of mesenchymal stem cells therapy in tumor microenvironment: pros and cons

**DOI:** 10.3389/fcell.2023.1255697

**Published:** 2023-10-02

**Authors:** Hamed Afkhami, Golnaz Mahmoudvand, Arshia Fakouri, Alireza Shadab, Mohamad Mahjoor, Tahereh Komeili Movahhed

**Affiliations:** ^1^ Nervous System Stem Cells Research Center, Semnan University of Medical Sciences, Semnan, Iran; ^2^ Cellular and Molecular Research Center, Qom University of Medical Sciences, Qom, Iran; ^3^ Department of Medical Microbiology, Faculty of Medicine, Shahed University, Tehran, Iran; ^4^ Student Research Committee, USERN Office, Lorestan University of Medical Sciences, Khorramabad, Iran; ^5^ Department of Immunology, School of Medicine, Semnan University of Medical Sciences, Semnan, Iran; ^6^ Iran University of Medical Sciences, Deputy of Health, Tehran, Iran; ^7^ Department of Immunology, Faculty of Medicine, Iran University of Medical Sciences, Tehran, Iran

**Keywords:** mesenchymal stem cell (MSC), cell-and tissue-based therapy, stem cell transplantation, neoplasm, tumor microenvironment (TME)

## Abstract

Multipotent mesenchymal stem cells (MSCs) are widely accepted as a useful tool for cell-based therapy of various diseases including malignancies. The therapeutic effects of MSCs are mainly attributed to their immunomodulatory and immunosuppressive properties. Despite the promising outcomes of MSCs in cancer therapy, a growing body of evidence implies that MSCs also show tumorigenic properties in the tumor microenvironment (TME), which might lead to tumor induction and progression. Owing to the broad-spectrum applications of MSCs, this challenge needs to be tackled so that they can be safely utilized in clinical practice. Herein, we review the diverse activities of MSCs in TME and highlight the potential methods to convert their protumorigenic characteristics into onco-suppressive effects.

## Graphical Abstract

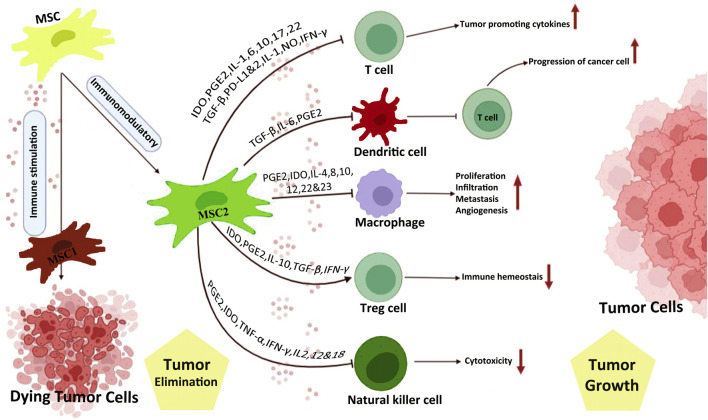

## Introduction

Cancer is the major obstacle to improving life expectancy in the 21st century (Moslemi et al., 2021; Nagy et al., 2023). The morbidity and mortality of cancer are rising expeditiously because of aging and population growth (Sohrabi et al., 2021; Bisht et al., 2023). Cancer therapy is among the most crucial clinical challenges. Surgical intervention and chemotherapy, as the most common therapeutic methods, may be associated with different complications (Debela et al., 2021; Zeng et al., 2023). Despite the development of different therapy methods, metastatic tumors are mainly untreatable and are responsible for the preponderance of deaths due to cancer (Espona-Fiedler et al., 2023). A major barrier to the development of efficient therapies is the complexity of tumors. The tumor heterogeneity increases as cancer progresses and the components of the tumor microenvironment (TME) become fully developed. The TME contains extracellular matrix and stromal cells, as well as immune cells, thereby playing a substantial role in the evolution of malignant tumors (Roma-Rodrigues et al., 2019). In recent years, novel therapeutic approaches including stem cell therapy, targeted therapy, nanoparticles, ablation therapy, radionics, natural antioxidants, and chemodynamic therapy have been introduced. These methods have improved the outcomes of patients, nevertheless, further advancements in drug delivery systems are required to refine therapeutic outcomes (Debela et al., 2021).

Recently, mesenchymal stem cells (MSCs) have been of great interest in the field of cancer therapy. MSCs are precursor cells that have the ability to self-regulate and proliferate. Under particular circumstances, they can differentiate into numerous mesenchymal tissues (Chen et al., 2019; Mahjoor et al., 2021). MSCs are obtained from different tissues, such as bone marrow, adipose tissue, skin, salivary gland, limb buds, dental tissues, menstrual blood, and placenta. MSCs are primarily isolated as plastic-adherent cells via tissue mincing, enzymatic digestion, and cell outgrowth. The most commonly used procedures are enzymatic and explant techniques. In the explant protocol, the source tissue is rinsed and cut into small fragments. Afterward, the tissue fragments are transferred to plastic culture vessels containing growth medium. In the enzymatic technique, tissue pieces are incubated with enzymes that degrade the extracellular matrix (Mushahary et al., 2018; Mahjoor et al., 2022). MSCs express specific adhesion molecules (e.g., CD13, CD29, CD44, CD49b, CD58, CD73, CD105, and CD166). Besides, MSCs derived from different sources express specific markers. For instance, CD29 and CD49b are mainly expressed by MSCs isolated from the placenta, while bone marrow-MSCs (BM-MSCs) demonstrate higher levels of CD90 (Montesinos et al., 2009).

Immunomodulatory properties of MSCs are mediated by various cytokines, including transforming growth factor (TGF-β), hepatic growth factors (HGF), prostaglandin E2 (PGE2), interleukins (Khakoo et al., 2006), indolamine 2,3-dioxygenase (IDO), and nitric oxide (NO) (Soleymaninejadian et al., 2012; Mahmoudvand et al., 2023; Mirshekar et al., 2023). MSCs are divided into various subtypes, which show different features, accordingly can both boost and suppress tumor progression by exerting influence on tumor cells through different mediators and intercellular interactions as well as adjusting the innate and acquired immune response (Galland and Stamenkovic, 2020; Janmohammadi et al., 2023). MSC1 and MSC2 are two important phenotypes of MSCs. The former shows pro-inflammatory properties while the latter exerts immunosuppressive effects. Strong evidence confirms that MSC1 is primarily anti-tumorigenic, while MSC2 favors tumor cell growth. Tumor growth-promoting effects of MSCs include expression of growth factors, improvement of tumor angiogenesis, and formation of tumor stem cell micro-environment (Ramdasi et al., 2015). On the other hand, antitumorigenic effects of MSCs are exerted through several pathways including, promotion of the immune response, suppression of angiogenesis, control of cellular signaling, and induction of cancer components apoptosis (Atiya et al., 2020). In this review article, the anti-tumorigenic and protumorigenic properties of MSCs will be highlighted first and we further discuss the solutions that have been proposed to eliminate the protumorigenic activity of MSCs and convert them to anti-cancer features.

## MSCs and anti-tumor properties

Investigations have revealed that despite the positive impact of MSCs on tumorigenesis, they can limit tumor growth. These effects may be exerted via different mechanisms.

• **Effects of MSCs on TME**

**FIGURE 1:**
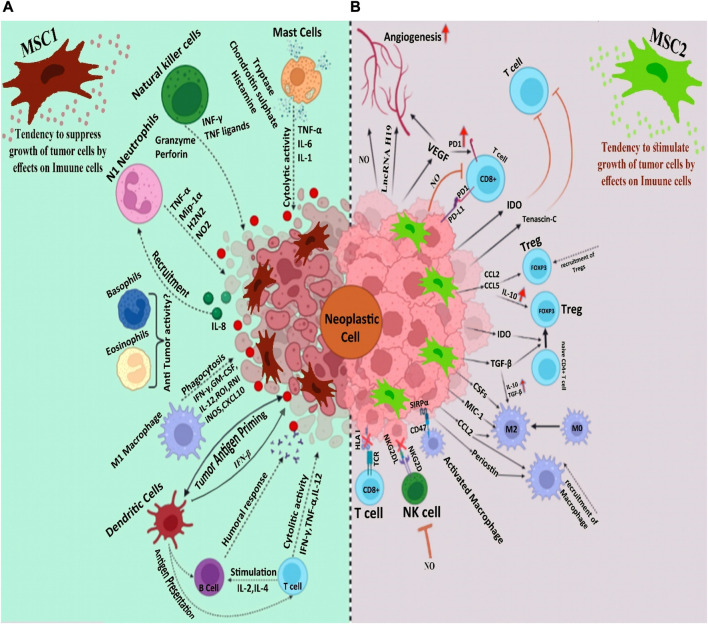
**(A)**. Mesenchymal stem cells strengthen the body’s immune system to combat tumors **(B)**. The immune system, with its immunomodulatory and anti-inflammatory properties, shuts down the immune system, as a result of which we see the progress of the tumor.

Via their strong proinflammatory properties, the combination of MSCs and tumor cells enhances the infiltration of monocytes, granulocytes, and T lymphocytes. The elevated infiltration of inflammatory cells facilitates interaction between immune cells and the adjacent tissues. These immune cells and the inflamed tissues enclosing them can produce several chemokines that recruit activated lymphocytes with their correlating receptors, hence provoking antitumor immunity (Figure 1A) (Ohlsson et al., 2003). Researchers have reported that iNOS-expressing MSCs successfully hinder the growth of fibrosarcoma cells (Xiang et al., 2009). As a matter of fact, iNOS synthesized by stromal cells plays a dual role in TME. M1 and M2 macrophages are essential determiners in the early and late stages of tumor growth (Trivanović et al., 2016). A similar behavior can be attributable to MSCs. Despite the lack of convincing evidence for MSC’s involvement in M1 polarization, the existence of iNOS-expressing M1 macrophages in tumor milieu suggests that MSCs may have the ability to acquire an M1 phenotype. Hence, it is reasonable to conclude that iNOS acts as a switch molecule of phenotypes of MSCs and macrophages in the tumor milieu. Overall, these findings point to the intricate cross-talks between macrophages and MSCs in TME (Sainz et al., 2016).

• **Antitumor properties exerted through signaling pathways**

Khakoo and co-authors (Khakoo et al., 2006) found that MSCs inhibited tumor progression *in vivo* by reducing target cell AKT activation in Kaposi’s sarcoma (KS). Nevertheless, they observed that when KS tumor cells were modified to express active AKT constantly, KS tumors were no longer susceptible to MSC treatment. These results imply that MSCs produce significant antitumorigenic properties via blocking AKT signaling. Furthermore, others have found that MSCs decrease breast cancer cell growth through the Wnt pathway, which is critical in oncogenesis (Qiao et al., 2008). MSCs have been administered systemically to deliver a binary vector containing an OAd along with a helper-dependent Ad that expresses IL-12 and programmed death-ligand 1 blocker (PD-L1). These MSCs deliver and synthesize viruses to invade and destroy lung tumor cells while triggering the onco-suppressive properties of chimeric antigen receptor-T (CAR-T) cells by producing IL-12 and PD-L1 blockers. *In vivo*, administration of combinatorial Ad vector MSCs causes a more significant rise in the number of T cells compared to CAR-T cells and propagates their polyfunctional cytokine release (McKenna et al., 2021). Moreover, in a study by Lu et al. (Lu et al., 2008), In cancerous cells, injection of MSCs increased the messenger ribonucleic acid (mRNA) expression of p21 (cell cycle negative regulator) and caspase 3 (apoptosis-related protease). Their results indicated that MSCs may suppress the growth of cancer *in vitro* and *in vivo* by enhancing cancer cell apoptosis and G0/G1 phase arrest. In addition, research has demonstrated that MSCs control cancer by decreasing tumor angiogenesis employing endothelial cell death and capillary degeneration (Otsu et al., 2009). As shown in a study, bone marrow MSCs inhibited vascular development in 1Gli36 glioma xenografts through suppression of the platelet-derived growth factor/platelet-derived growth factor receptor (PDGF/PDGFR) axis. Particularly, the expression of PDGF-BB protein considerably decreased in tumor lysates when treated with MSCs, which was associated with diminished concentrations of activated PDGFR-b and its subsequent target AKT isoform (Ho et al., 2013a). Lately, Gu and co-authors (Gu et al., 2021) discovered that MSCs-derived exosome could suppress hepatocellular cancer stem cells (CSCs) malignancy through a long noncoding RNAs (lncRNAs) C5orf66-AS1/micro-RNA-127-3p/dual-specificity phosphatase 1 (DUSP1)/ERK axis. Given the role of exosomes in both tumorigenic and anti-tumor activities of MSCs, hepatocellular CSCs were treated with exosomes, leading to a marked decrease in the proliferation, migration, invasion, angiogenesis-inducing, and self-regeneration capacities of CSCs via lncRNA C5orf66-AS1/microRNA-127-3p/DUSP1 axis and blockage of the phosphorylation of ERK *in vitro*. Similarly, *in vivo* investigation revealed that exosomes diminished the growth of xenografts made by CSCs in nude mice (Xuan et al., 2021). MSCs produce cytotoxic factors, including TNF-related apoptosis-inducing ligand (TRAIL) that selectively drives apoptosis in several types of malignancies (Hao et al., 2001; Takeda et al., 2001). *In vivo* investigations in the murine xenograft model of intraperitoneal human mesothelioma revealed the potential of TRAIL-expressing MSCs for dampening inflammatory responses in TME (Lathrop et al., 2015). Findings of another study conducted in 2019 showed that bone marrow MSCs could enhance apoptosis and inhibit the progression of glioma U251 cells via downregulating the PI3K/AKT signaling cascade (Lu et al., 2019). IFN-β-releasing BM-MSCs have been observed to diminish the growth of hepatocellular carcinoma cells primarily by affecting their cell cycle, reducing the expression of cyclin D1 and phosphorylation of Rb via silenced Akt and promoting FOXO3a activity (Xie et al., 2013).

• **Cytokine-mediated mechanisms**

IL-18-overexpressing umbilical cord MSCs (UC-MSCs) have been shown to attenuate the growth and spread of breast cancer cells, probably by modifying the cell cycle of cancerous cells (Liu et al., 2015). Additionally, by producing inflammatory cytokines such as the multifunctional cytokine TGF-β, MSCs have been shown to trigger anti-tumor immune responses. TGF-β signaling has inhibitory effects in cancer. Although the expression of the type III TGF-β receptor (TbRIII) drops throughout the evolution of breast cancer, restoring TbRIII expression inhibits tumorigenicity. This is even though TbRIII expression drops during progression. (Dong et al., 2007; Guasch et al., 2007) (Table 1).

**TABLE 1:** The role of MSCs in tumor suppression.

MSC type	Factors	Mechanisms	Outcome
BM-MSCs	CCL-7 and CCL-12	BM-MSCs treated with TNF-α increase the recruitment of monocytes, macrophages, and neutrophils to the tumor via CCL-7 and CCL-12	Boosting monocyte and granulocyte infiltration (Ren et al., 2012)
BM-MSCs	TGFβ and TFF3	MSCs modulate the inflammatory response during the early phase of carcinogenesis through TGFβ and TFF3	Suppression of tumor cell progression (François et al., 2018)
BM-MSCs	Wnt/β-catenin	MSC-derived exosomes reduce tumor development by distorting the Wnt/β-catenin signaling pathway	Suppression of tumor cell progression (Xu et al., 2019)
hAMSCs	E-cadherin	MSC induces upregulation of E-cadherin	Suppression of EMT (Safari et al., 2021)
BM-MSCs	PDGF and IL-1β	BM-MSC inhibits the release of antiangiogenic factors, including PDGF and IL-1β	Inhibition of angiogenesis (Ho et al., 2013b)
hAMSCs	Bax and caspase-3	MSC induces the expression of Bax and caspase-3 in tumor cells	Induction of tumor cell apoptosis (Safari et al., 2021)
UC-MSCs	PI3K/AKT and JNK	MSCs induce apoptosis in cancer cells via downregulation of PI3K/AKT and activation of JNK signaling	Induction of tumor cell apoptosis (Han et al., 2018)
T-MSCs	Bax, p53, c-myc	MSC triggers the upregulation of Bax, p53, and c-myc genes in tumor cells	Induction of tumor cell apoptosis (Yüce and Albayrak, 2021)

BM-MSCs , bone marrow-derived mesenchymal stem cell; TNF-α , tumor necrosis factor *α*, CCL-7, chemokine ligand 7; CCL-12, chemokine ligand 12; TGFβ, transforming growth factor beta, TFF3 = trefoil factor 3, hAMSC , human amniotic mesenchymal stromal cell; EMT , epithelial-mesenchymal transition; PDGF, platelet-derived growth factor, IL-1β = interleukin 1β, Bax = Bcl-2-associated X protein, UC-MSCs, umbilical cord-derived mesenchymal stem cell, PI3K/AKT, phosphatidylinositol 3-kinase/Akt, JNK = c-Jun N-terminal kinases, T-MSC, tonsil-derived mesenchymal stem cell.

## Immunosuppression and tumorigenesis features of MSCs

• **Tumorigenic effects of MSCs on TME**

Heterogeneity within the TME is an important factor with a detrimental effect on the development of tumors. The TME is an intricate environment, composed of stromal cells and components of the extracellular matrix, along with secreted factors (Nilendu et al., 2018). Stromal cells in TME combine endothelial cells, adipocytes, cancer-associated fibroblasts, immune cells, and MSCs (Spaw et al., 2017; Tao et al., 2023). Notably, MSCs exhibit significant tropism to tumor sites, which may either speed up or slow down the progression of cancer (Xuan et al., 2021). Toll-like receptors (TLRs) exist in MSCs, among other cell types. TLRs are able to recognize signals of ‘danger,’ and once they are activated, a wide range of cells, particularly immune cells and MSCs, are drawn to the injury site. It is noteworthy that activation of TLR3 drives MSCs to produce factors that primarily have an immunomodulatory effect on the tumor cells (such as IL-1 receptor antagonist and IL10), whereas activation of TLR4 results in the production of inflammatory and proapoptotic factors by MSCs (such as IL17, granulocyte-macrophage colony-stimulating factor, and TRAIL). Degrading tryptophan is another process through which IDO synthesized by MSCs was able to block allogeneic T-cell responses (Meisel et al., 2004). In particular, in naive CD4+T cells, tryptophan catabolism induced the production of the forkhead box P3-positive regulatory T cells (Fallarino et al., 2006). These cells impeded the responses of effector T cells, which led to a decrease in anti-tumor immunity. Current research has introduced an innovative method through which MSCs control the activity of the immune system. In fact, MSCs attracted myeloid-derived suppressor cells (MDSCs) in a C-C motif chemokine ligand 2 (CCL2)-dependent mechanism, hence lowering the activity of anti-cancer T cells even further (Lee et al., 2015). The MDSC is considered the main protector of the TME, providing an immunosuppressive shield that protects the cancerous cells from the host’s immune system (Tesi, 2019). MSCs have the ability to decrease the activities of both T cells and B cells as part of the adaptive immune response. MSCs were able to restrict B cell activity by producing humoral chemicals via reducing B cell terminal differentiation (Asari et al., 2009). Overall, MSCs have powerful inhibitory effects on adaptive immune response, which can be used by cancer cells inside TME. MSCs not only suppress the adaptive immune response, but they also inhibit the innate immune cells, causing a reduction in the effectiveness of the basic immunological responses against cancer. MSCs inhibited the formation and function of monocyte-derived DCs, causing a reduction in the expression of the costimulatory molecules CD80 and CD86. This resulted in a limitation of the allogeneic T cell’s potential for allostimulation (Jiang et al., 2005). Importantly, macrophage functioning in the TME was directly suppressed by MSCs. It has been reported that the conditioned medium formed from MSC may inhibit the phagocytic activity of macrophages, hence further lowering anti-cancer immunity (Chen et al., 2018). Moreover, the activity of neutrophils was affected by the presence of MSCs. In a model of breast cancer, CD11b+Ly6G + neutrophils were cocultured with MSCs and then taught to obtain immunomodulatory properties. This training resulted in the neutrophils inhibiting the proliferation of T cells *in vitro* and accelerating tumor growth *in vivo* (Hu et al., 2014). The mesenchymal niche might also be implicated in cancer metastasis. Growing shreds of evidence show that MSCs have the capacity to migrate to tumor locations, including both primary and pre-metastatic sites (Kaplan et al., 2005). Tumor-secreted elements might move to surrounding tissues (Bergfeld and DeClerck, 2010) and draw MSCs to aid in the formation of mesenchymal niche, propagating tumor cell migration. Breast cancer cells promote the synthesis of CCL5 (also called RANTES) from MSCs by communicating with C-C chemokine receptor type 5, enhancing cancer cell motility, invasion, and distant spread *in vitro* and *in vivo* (Karnoub et al., 2007). Once affected by oxidative stress in the TME, MSCs can release lactate, and when lactate is absorbed by cancer cells, they can migrate more efficiently by producing ATP (Bonuccelli et al., 2014). In particular, MSCs were found to differentiate into cancer-associated fibroblasts *in vitro*, which promotes tumor heterogeneity and aids in cancer growth and drug resistance (Miyazaki et al., 2020). Several researchers have also shown that noncoding RNAs are involved in tumorigenesis and drug resistance (Asari et al., 2009; Wang et al., 2015; Yuan et al., 2016). Taken together, the evidence revealed the role of MSCs in boosting cancer progression via different mechanisms, hence targeting MSCs can be a potential strategy for cancer therapy (Xuan et al., 2021).

• **Cytokine-mediated mechanisms**

MSCs were found to block the oncosuppressive innate and adaptive immune responses, via releasing several soluble factors and mediators (e.g., PGE2, interferon-gamma (IFNγ), IL-4, indoleamine 2,3-dioxygenase (Kaplan et al., 2005), TGF-β1, IL-6) and cross-talking with a wide range of immune cell types (e.g., T cell, B cells, macrophages, dendritic cells, natural killer (NK) cells, and neutrophils) (Rivera-Cruz et al., 2017). MSCs inhibit both the activation and proliferation of T cells, which serve a substantial role in adaptive immunological responses. PGE2 is released by MSCs, which subsequently binds to prostaglandin EP2 and EP4 receptors on macrophages, causing them to produce the anti-inflammatory cytokine IL-10 and limit T cell activity (Németh et al., 2009). Besides, T helper 2 (Th2)-polarized immune response is evoked by MSCs. In fact, they cause a reduction in inflammatory T cells and their related cytokines (Th1 cells-IFNγ), while elevating anti-inflammatory T cells and related cytokines (Th2 cells-IL4) (Bai et al., 2009). MSCs were also able to suppress T cell activation via secreting immunosuppressive TGF-β1, which adheres to glycoprotein A repetitions predominant (GARP) located on MSCs (Niu et al., 2017). IFNγ-activated MSCs were accompanied by an upregulation in the expression of galectin-9, resulting in suppressed antigen-driven immunoglobulin secretion and lowered B cell proliferation (Ungerer et al., 2014). The functions of NK cells are inhibited by MSC-originated PGE2 and IL-6. Moreover, MSCs were shown to largely suppress the synthesis of IFN-γ in NK cells, which reduced the anti-cancer efficacy of the NK cells (Galland et al., 2017). Dendritic cells (DCs), which play a role in the process of presenting antigens, are intricately associated with anti-cancer activity. It has been shown that the maturation and function of DCs were impeded when PGE2 produced by MSCs was present in the environment (Spaggiari et al., 2009). Also, MSC-derived PGE2 stimulated a switch from inflammatory M1 macrophages to a pro-tumorigenic M2 state, which was associated with increased concentrations of immune-inhibitory IL-10 (Vasandan et al., 2016). The aforementioned evidence suggests that MSCs are able to inhibit the immune response to tumors, which in turn promotes the progression of tumors. In addition, MSCs exhibited the ability to promote the proliferation of cancer cells as well as neovascularization. In breast and prostate cancers, for example, MSCs increased the levels of pro-angiogenic factors such as vascular endothelial growth factor (VEGF), macrophage inflammatory protein-2 (MIP-2), TGF-β, and IL-6. Owing to the effects of these substances, tumor proliferation and angiogenesis were triggered, thereby solid tumor formation was sped up both *in vitro* and *in vivo* (Zhang et al., 2013a). Tumor cell apoptosis is also inhibited by MSCs. Hypoxia, malnutrition, and inflammation all contribute to tumor pathogenesis. Under this circumstance, MSCs maintain their self-survival via autophagy and secreting a variety of pro-survival or anti-apoptotic factors, such as basic fibroblast growth factor (bFGF), PDGF, VEGF, TGF-β, stromal cell-derived factor 1 alpha (SDF-1α), NO, and hepatocyte growth factor (HGF) (Hung et al., 2007). As an illustration, vascular VEGFs and bFGF, can promote Bcl-2 expression (König et al., 1997; Dias et al., 2002); on the other hand, PDGF and TGF-β upregulate VEGF and bFGF gene expression (Brogi et al., 1994). SDF-1α is able to defend leukemia cells against spontaneous apoptosis (Burger et al., 2000), and HGF improves the angiogenic and anti-apoptotic effects (Efimenko et al., 2011). Besides, NO has been proposed to act as a dual-function apoptotic regulator; At large doses, NO exerts proapoptotic effects, while at low doses, it has antiapoptotic function (Stamler, 1994) (Table 2).

• **Anti-tumor effects via signaling cascades**

**TABLE 2:** The role of MSCs in enhancing tumor progression.

MSC type	Factors	Mechanisms	Outcome
BM-MSCs	SphK1	SphK1 in BMSCs is triggered by TGF-β1 resulting in differentiation of BMSCs into myofibroblasts via S1PR1 and S1PR3 upregulation	Differentiation into cancer-associated fibroblasts (Yang et al., 2012)
UC-MSCs	IL-6 and HGF	UC-MSCs produce IL-6 and HGF and induce the synthesis of IL10, which is involved in immune suppression	Modulation of the anti-tumor immune responses (Deng et al., 2016)
BM-MSCs	NO and PGE2	NO synthesized by MSC and PGE2 contributes to the inhibition of T cells	Modulation of the anti-tumor immune responses (Sato et al., 2007)
BM-MSCs	IDO	MSCs express IDO protein that inhibits allogeneic T-cell responses	Modulation of the anti-tumor immune responses (Meisel et al., 2004)
BM-MSCs	TGF-β, LIF, TSG-6, COX-2, PD-L1, IL-8, CCL2	The molecules exert immunoregulatory function, including induction of T-cells to differentiate into anti-inflammatory phenotypes	Modulation of the anti-tumor immune responses (Svobodova et al., 2012; Holan et al., 2018)
BM-MSCs	Twist, Snail, FOXC2	These factors produced by MSC promote epithelial-mesenchymal transition (EMT), which can enhance cancer progression	Promotion of the EMT (Battula et al., 2010)
BM-MSCs	IL-6 and JAK2/STAT3	Secretion of IL-6 by MSCs triggers JAK2/STAT3 cascade activation	Enhancement of cancer cell stemness (Hsu et al., 2012)
		in cancer cells, leading to the enhancement of tumor formation	
BM-MSCs	IL6 and CXCL7	MSC regulates cancer stem cells via IL6 and CXCL7	Enhancement of cancer cell stemness (Liu et al., 2011)
BM-MSCs	TGF-β, VEGF, IL-6, and MIP-2	MSC produces pro-angiogenic factors when exposed to tumor cells	Reinforcement of tumor angiogenesis (Zhang et al., 2013b)
BM-MSCs	IL-6, STAT3, MRP, and MDR-1	MSCs exert chemoprotective effects via IL-6, which is activated by STAT3, MRP, and MDR-1	Enhancement of cancer cell survival (Tu et al., 2016)
BM-MSCs	STC1 and UCP2	STC1 derived by MSC upregulates UCP2 resulting in increased cancer cell survival	Enhancement of cancer cell survival (Ohkouchi et al., 2012)
BM-MSCs	CCL5	Cancer cells stimulate the secretion of CCL5 from MSCs leading to the elevated invasion and metastasis of tumor	Augmentation of tumor invasion and metastasis (Karnoub et al., 2007)

BM-MSC, bone marrow-derived mesenchymal stem cell, SphK1 = sphingosine kinase 1, TGF-β, transforming growth factor-β, S1PR1 = sphingosine 1-phosphate receptor 1, S1PR3 = sphingosine 1-phosphate receptor 3, UC-MSC, umbilical cord-derived mesenchymal stem cell; IL, interleukin; HGF, hepatic growth factor; NO, nitric oxide, PGE2 = prostaglandin E2, IDO, indolamine 2,3-dioxygenase, LIF, leukocyte inhibitory factor; TSG-6 , tumor necrosis factor a-stimulated gene 6, COX-2, cyclooxygenase-2; PD-L1, programmed death ligand 1, CCL2 = chemokine ligand 2, FOXC2 = mesenchyme forkhead 1, JAK2/STAT3 = Janus kinase 2/signal transducer and activator of transcription 3, CXCL7 = chemokine ligand 7, VEGF, vascular endothelial growth factor; MIP-2 , macrophage inflammatory protein 2; MRP , multidrug resistance protein; MDR-1 , multidrug resistance p-glycoprotein, STC1 = secretion of stanniocalcin-1, UCP2 = upregulated uncoupling protein 2, CCL5 = chemokine ligand 5.

In a gastric cancer model, chemotaxis, survival, and stimulation of neutrophils were modulated by IL6-STAT3-ERK1/2 signaling (Zhu et al., 2014). In a hepatocellular carcinoma model, Li and colleagues observed a remarkable increase in the microvessel density and TGFβ1 mRNA levels, as well as a noticeable reduction in Smad7 mRNA in the subjects treated with MSC. These findings insinuated that MSCs might serve pro-angiogenic effects via the TGFβ1/Smad pathway (Li et al., 2016). Similarly, in a gastric cancer model, TGF-β1 secreted by MSCs stimulated the SMAD2/3 pathway and enhanced tumor growth via the lncRNA MACC1-AS1/miR-145-5p/fatty acid oxidation axis in cancer cells (He et al., 2019). Yuan et al. also discovered that LncRNA H19 contributes to MSC-mediated angiogenesis (Yuan et al., 2019a). Their results pointed to the fact that LncRNA H19 knockdown in MSCs blocked neovascularization by interacting with histone methyltransferase EZH2 and inducing the angiogenesis inhibitor gene VASH1, diminishing angiogenesis factors release, and promoting the formation of angiogenesis inhibitors. Importantly, MSCs can induce the spread of cancerous cells; Breast cancer cells treated with MSCs showed overexpression of oncogenes (NCOA4, FOS), proto-oncogenes (FYN, JUN), and EMT-specific markers, leading to breast cancer metastasis (Martin et al., 2010). MSCs also increase tumor growth by modifying their metabolic state. In lymphoblastic leukemia, MSCs-derived PGE2 activated cAMP-PKA signaling in tumor blasts and blocked the antitumor role of wild-type p53, thus promoting leukaemogenesis (Naderi et al., 2015) (Table 3).

**TABLE 3:** Overview of the anti-tumorigenic behavior of different types of MSCs.

MSC origin	Type of cancer	Anti-tumorigenic effects
BM-MSC	**Kaposi’s sarcoma**	Intravenously injection of BM-MSC suppressed tumor development in a mouse model of Kaposi’s sarcoma (Khakoo et al., 2006)
	**Colon cancer**	The use of BM-MSC resulted in cytotoxicity against colon cancer cell lines (Larmonier et al., 2003)
	**non-Hodgkin’s lymphoma**	BM-MSC showed anti-tumor activity against disseminated non-Hodgkin’s lymphomas in a mouse model (Secchiero et al., 2010)
	**Kidney**	BM-MSC reduced the growth of renal cell carcinoma and improved survival via releasing IL-12 (Gao et al., 2010)
	**Liver**	Systemically administered measles virus-infected BM-MSCs inhibited liver cancer growth (Ong et al., 2013)
AT-MSC	**Brain**	The injection of AT-MSC-HSV-Tk cells combined with ganciclovir caused a significant decrease in glioblastoma cells in nude mice (de Melo et al., 2015)
	**Breast**	AT-MSC increased chemosensitivity of human breast cancer cells SKBR3 (Kucerova et al., 2013)
	**Prostate**	Administration of AT-MSCs into mice treated with 5-FC led to a complete tumor regression (Cavarretta et al., 2010)
	**Pancreas**	Suppressed pancreatic ductal adenocarcinoma proliferation, both *in vitro* and *in vivo,* and promoted tumor cell death via modifying cell cycle progression (Cousin et al., 2009)
UC-MSC	**Breast**	UC-MSC-derived exosomes carrying miRNA-148b-3p suppressed breast cancer progression (Yuan et al., 2019b)
	**Lung**	Silencing TGF-β1 expression enhances the pro-apoptotic effects of MSC-exosome on lung cancer cells (Zhao et al., 2018)
	**Prostate**	UC-MSCs drive apoptosis in PC-3 prostate cancer cells via downregulation of PI3K/AKT and activation of JNK signaling (Han et al., 2018)
hAMSCs	**Prostate**	The anti-tumor effects of hAMSCs on LNCaP prostate cancer cells through induction of apoptosis, suppression of epithelial-mesenchymal transition process, and downregulation of EGFR were shown (Safari et al., 2021)
	**Bladder**	MSC-derived exosomal miRNA-139-5p showed onco-suppressive activities in bladder cancer (Jia et al., 2021)

BM-MSC, bone marrow-derived mesenchymal stem cells; AT-MSC, adipose tissue-derived mesenchymal stem cells; UC-MSC , umbilical cord-derived mesenchymal stem cells; miRNA , micro ribonucleic acid; TGF-β1 , transforming growth factor beta 1; hAMSCs, human amniotic mesenchymal stem cells; EGFR , epidermal growth factor receptor.

## Are there ways to convert the tumorigenic properties of MSCs to anti-tumorigenic?

As mentioned before, the role of MSCs in cancer progression is controversial. *In vivo* and *in vitro* studies have demonstrated that MSCs have the ability to suppress tumor growth. However, there is robust evidence that confirms the substantial role of MSCs in promoting cancer and metastasis through various pathways (Atiya et al., 2020; Bui et al., 2023). Since decades ago, various therapeutic advantages have been proposed for exogenous MSCs. Application of MSCs in the field of tissue regeneration has shown promising outcomes in the treatment of cardiovascular diseases, stroke, lung disorders, renal failure, rheumatic diseases, neurological disorders, *etc.* Nevertheless, emerging evidence on the tumorigenic function of MSCs raises concerns about their safety in clinical applications (Galland and Stamenkovic, 2020). As reported in an article, a boy diagnosed with ataxia-telangiectasia who received human fetal neural SCs developed a glioneuronal tumor 4 years after the first SC- injection. Further assessment revealed that the tumor was of non-host origin, implying that the tumor arose from the transplanted neural SCs (Amariglio et al., 2009). The findings of another survey suggested that chronic infection of C57BL/6 mice with *Helicobacter* triggers repopulation of the stomach with BM-MSCs, which then undergo metaplasia and dysplasia to induce intraepithelial cancer (Houghton et al., 2004). These and other similar reports persuaded scientists to find methods for enhancing the anti-tumor properties of MSCs relative to their tumorigenic activities and converting them into unquestionable therapeutic agents (Liang et al., 2021).

• **Genetic modification of MSCs**

Tumor specificity is the main barrier to the effectiveness of conventional cancer therapy. MSC’s tendency towards tumor sites improves drug specificity by resolving the issues of stability, dosing, and toxicity related to systemic administration of drugs. This approach has been previously used for the controlled release of anti-cancer agents and has shown promising results when using genetically modified MSC (GM-MSC) against various cancers in animal models (Hagenhoff et al., 2016; Christodoulou et al., 2018). Several studies used GM-MSCs as a tool to transfer and express different onco-suppressive agents such as IFN α and *β*, IL-2, IL-12, CXCL1, TRAIL, and oncolytic virus. Since GM-MSCs elevate the local concentration of these agents, their anti-tumorigenic function is more effective relative to their function when applied systematically. Furthermore, manipulated MSCs can express certain enzymes that may modify inactive systemically used prodrugs such as ganciclovir into active cytotoxic medications (Liang et al., 2021). von Einem et al. investigated the efficacy of autologous GM-MSC combined with ganciclovir in the treatment of advanced gastrointestinal adenocarcinoma. The results showed that this combination was tolerable and safe which led to clinical stabilization of malignancy and a higher overall survival rate than expected in patients (von Einem et al., 2019). Other studies have similarly reported the therapeutic effectiveness of GM-MSCs against lung, brain, and breast cancers (Christodoulou et al., 2018). By way of illustration, Fei et al. investigated the effects of cytosine deaminase-expressing MSCs in a rat model of C6 glioma. This strategy reduced the tumor volume, propagated tumor cell apoptosis, and improved the survival time (Fei et al., 2012). Gene-directed enzyme/prodrug therapy using adipose MSCs that expressed herpes simplex virus thymidine kinase (TK) demonstrated a great potential for glioblastoma therapy. A group of researchers showed that canine adipose MSCs can be treated with a lentiviral vector to express TK. Combined with ganciclovir, this prodrug exerted antitumor effects on human glioblastoma cell line U87 in a murine model (Villatoro et al., 2022). Adipose SCs were also genetically modified to express recombinant secretory human carboxylesterase-2 and nanoluciferase genes. These cells effectively targeted and localized at tumor stroma and necrotic tissues, and when used together with irinotecan, destroyed all intraperitoneal tumor cells and ameliorated the survival (Malekshah et al., 2019). In another experiment, the combination of the suicide gene CYP2B6TM-RED (a fusion of a triple mutant of CYP2B6 with NADPH cytochrome P450 reductase) and cyclophosphamide showed promising results in treating solid tumors. MSCs as cellular vehicles for the delivery of our suicide genes. MSCs expressing CYP2B6TM-RED could activate cyclophosphamide and eliminate the surrounding tumor cells (Amara et al., 2016).

• **Preconditioning with pro-inflammatory cytokines**

Macrophages, which can be present as pro-inflammatory M1 and alternatively activated M2 cells, contribute to different inflammatory responses. MSCs can steer monocytes to differentiate into anti-inflammatory M2 phenotypes. Thus, MSCs have the ability to inhibit excessive immune response. MSCs feature immunosuppressive effects that need to be promoted by supportive signals. The immunosuppressive properties of MSCs can be affected by certain pro-inflammatory cytokines IFN-γ, TNF-α, and IL-1α. The stimulation of MSCs by these cytokines is essential for the demonstration of their immunosuppressive behavior (Kim et al., 2018a). Philipp and colleagues found that MSCs preconditioned with IL-1ß and IFN- γ released high amounts of PGE2, NO, and IL-6. Additionally, co-culture with M0 macrophages under the influence of M1 inducers, lipopolysaccharide, and IFN-γ, caused a marked drop of CD86 and iNOS protein in macrophages and reduced TNF-α release. Overall, this method was highly effective in promoting the immunosuppression behavior of MSCs (Philipp et al., 2018). The elevated immunosuppressive activity of MSCs following POLY-IC stimulation that has been observed in some studies is also a promising approach to improve conventional SC-based therapies (Sangiorgi and Panepucci, 2016).

• **MSC-extracellular vesicles (MSC-EVs)**

Recently, the application of MSC-EVs has been suggested as a potential cell-free therapeutic agent (Galland and Stamenkovic, 2020). EVs are defined as heterogeneous vesicles that act as mediators of intercellular interaction through their loaded proteins, or nucleic acids. Although significantly smaller in size, MSC-derived EVs show most of the features of MSC. MSC-EVs excel in many ways such as *in vivo* stability and long half-life (Lai et al., 2019). They play a major role in TME communications and akin to MSCs, MSC-EVs may demonstrate both onco-suppressive and protumorigenic activities (Shojaei et al., 2019). Various researchers have proposed that the cell source can condition EV homing to particular sites and that their membrane could be manipulated to elevate tissue-specific targeting. Hence, MSC-EVs can be utilized as biocompatible tools to deliver mRNA, microRNA (miRNA), non-coding RNAs, prodrugs, and peptides to the desired cells (Galland and Stamenkovic, 2020; Guo et al., 2022). By way of illustration, a group of researchers evaluated the application of membrane surface manipulation along with targeting EVs for reinforced uptake in cardiac tissues affected by ischemia via admixture of tissue-targeting antibodies, fluorescent tags, and homing peptide surface cloaks. Their findings showed that EV targeting could be boosted both by a surface display and cloaking (Antes et al., 2018). The activities of MSC-EVs have been investigated in different types of malignant tumors and promising anti-tumorigenic effects have been observed. Del Fattore et al. (Del Fattore et al., 2015) investigated the effects of MSC-EVs on glioblastoma cells. The results showed that UC- and BM-MSC-EVs reduced cell proliferation and increased apoptosis of glioblastoma cells. Some researchers have demonstrated improved efficacy of EVs when applied in combination with gene therapy methods. Gene therapy can be a beneficial method by providing ways to control and correct gene expression. Small interfering RNA (siRNA) and miRNA are among the main molecules applied to trigger gene suppression (Hu et al., 2020). For instance, Dong and colleagues (Dong et al., 2019) enriched human umbilical cord MSCs (UC-MSCs) with siRNA-ELFN1-AS1 and observed that EVs from these treated cells could suppress colon adenocarcinoma cell proliferation and migration *in vitro*. Kamerkar and co-authors (Kamerkar et al., 2017) evaluated the impact of siRNA carried by exosomes against oncogenic KRAS in human pancreatic cancer. They observed that this strategy remarkably reduced mRNA levels and phosphorylated-ERK protein concentrations in PANC-1 cells. Another group of researchers reported that the administration of anti-miRNA via MSC exosomes targeting glioblastoma multiforme was effective in the restoration of chemosensitivity of multidrug-resistant cells (Munoz et al., 2013). MSC-EVs have also been suggested as an excellent vehicle for drug therapy against malignant cells. As an illustration, the use of UC-MSC-EVs loaded with Vincristine has led to a further increase in cytotoxicity against glioblastoma cells compared with both free drugs and intact EVs (Del Fattore et al., 2015). Hence, the use of EVs seems to be an effective approach to enhance the onco-suppressive effects of MSCs.

• **Manipulating the protumorigenic signaling pathways**

Therapeutic blockade of signaling cascade molecules involved in tumorigenesis is another approach to suppress protumorigenic activities of MSCs. In this regard, both chemical and herbal products have been suggested to hinder the protumorigenic properties of MSCs. To give an example, MSCs have been shown to induce ovarian carcinoma STAT3 signaling through IL6 and LIF. A group of researchers used Ruxolitinib to target this signaling and observed increased survival in subjects following this therapy (McLean et al., 2019). Recently, the usage of herbal products in the suppression of protumorigenic activities of MSCs has also gained attention. The evaluation of the function of curcumin in adjusting gastric cancer cells-derived MSCs mediated angiogenesis has shown that this product can inhibit angiogenesis via suppressing NF-κB/VEGF signaling (Huang et al., 2017). Treatment with Astragalus polysaccharide, a traditional Chinese herb, has led to a protective impact on morphological changes in BM-MSCs triggered by lung cancer cells (Zhang et al., 2019). Wensheng Zhuanggu Formula inhibits BM-MSC-induced EMT and metastasis in breast cancer via downregulating TGF-β1/Smads signaling (Ma et al., 2020). Similarly, ginseng extract has been shown to inhibit the invasion of colon cancer cells by suppressing ERK1/2 and NF-κB pathways (Kim et al., 2018b)

## Conflicts remaining to be resolved

As mentioned earlier, the influence of MSCs on the tumor milieu is extensive and occasionally paradoxical. The majority of studies that have shown antitumorigenic effects for MSCs have applied MSCs with no previous exposure to cancer cells. This may reflect that cancer-naïve MSCs and cancer-educated MSCs have different functions (Atiya et al., 2020). Moreover, the anti-cancer activity of different types of MSC has been investigated compared to each other. The results have shown that UC-MSCs have significantly higher onco-suppressive effects compared to BM- MSCs and adipose tissue-MSCs (Christodoulou et al., 2018). The complex cellular and molecular interplays between MSCs and the TME can also cause discrepancies in results. MSCs can migrate to tumors and differentiate into various types of cells, including tumor-associated MSCs and tumor-associated fibroblasts (Quante et al., 2011). Neoplasm-derived signals can affect the phenotype of the recruited MSCs, making them a component of tumor tissue; these MSCs carry characteristics that are different from tissue-associated MSCs and BM-MSCs (Zhao et al., 2020). MSCs that have been primed with TLR4 are referred to as MSC1 and display an antitumorigenic effect, while MSCs that have been primed with TLR3 are known as MSC2 and have a tumor-supportive role (Figure 1B) (Waterman et al., 2012). Ruth et al. found that MSC1 was able to suppress tumor progression, while tumor growth and metastasis were promoted by MSC2 both *in vivo* and *in vitro* (Waterman et al., 2010). It is interesting to note that the specific TLR agonist that MSCs are exposed to facilitates the shift between MSC1 and MSC2. To further clarify, TLR4 agonists polarize MSCs in the direction of the pro-inflammatory MSC1 population, which is essential for early injury responses. On the other hand, TLR3 agonist exposure would polarize MSCs towards the immunosuppressive MSC2 population, which is essential for facilitating tissue repair. This might partly clarify why MSCs have such a wide function in the treatment of different forms of cancer. Co-culture with MSCs causes more breast, pancreas, and ovarian tumor cell colonies and larger masses compared to untreated controls. MSC2 co-culture leads to the most expanded colonies. On the contrary, co-culture with MSC1 is accompanied by fewer cancer colonies and smaller masses. Overall, these findings insinuate that MSCs and MSC2 promote tumor progression, while MSC1 hurdles tumor cell growth (Waterman et al., 2012). This is partially true of MSC-EVs as well as MSCs. Since MSC-EVs are non-living components, they lack the ability to cause neoplasms. Nevertheless, they may exert an influence on tumor progression. The impact of MSC-EVs on tumor growth is a matter of debate. Some investigations reported that MSC-EVs dampened tumor growth; on the other hand, there are pieces of evidence for the implication of MSC-EV in tumor progression and spread. Interestingly, all of the MSC-EVs used in these surveys also originated from naïve MSCs. Where MSC exosomes synthesized by MYC-transformed MSCs, E1-MYC cells were used, no inhibitory or promoting effects were observed on tumor growth. These controversial findings may be justified by the heterogeneity of MSC sources, different methods used for EV isolation, or discrepancy in tumor models studied (Tan et al., 2021) (Table 4). Further studies may help to explain the intricate interactions between MSCs and tumor components.

**TABLE 4:** Comparison of the effects of naïve and educated MSCs on tumor cells.

Naïve MSCs	Educated MSCs
Modifying the ratio of T regulatory and myeloid-derived suppressor cells to CD8^+^ T cells by recruiting diverse immune cells into the TME, which leads to the onco-suppressive state (Zheng et al., 2016)	Suppression of the anti-tumor immune responses by releasing different factors, including IL-6 and HGF (Deng et al., 2016)
hAMSCs show anti-tumour effects on cancer cells through induction of apoptosis and suppression of EMT (Safari et al., 2021)	MSCs release pro-angiogenic factors, when stimulated by tumor cells, promoting tumor growth and angiogenesis (Zhang et al., 2013b)
Naïve BM-MSC expresses appropriate levels of miR-15a which is involved in tumor suppression (Roccaro et al., 2013)	Expression of miR-15a is reduced in BM-MSC–derived exosomes exposed to multiple myeloma (Roccaro et al., 2013)
Naïve MSCs express low levels of markers known as cancer-associated fibroblasts (Arena et al., 2018)	The expression of cancer-associated fibroblasts is significantly increased in tumor-exposed MSCs (Arena et al., 2018)

TME, tumor microenvironment; hAMSC, human amniotic mesenchymal stem cell; EMT, epithelial mesenchymal transition; IL-6, interleukin 6; HGF, hepatic growth factor.

## Conclusion

MSCs can be considered the major regulators of tissue homeostasis. Evaluating the level of the inflammatory response to injury, MSCs can adapt effective functions to suppress or promote the response. Pro-tumorigenic effects of MSCs are exerted through various mechanisms in the TME, including differentiation into stromal components of the TME, suppression of immune response, enhancement of angiogenesis, improving tumor cell survival, and promotion of metastasis. On the other hand, many studies have suggested that MSCs have the potential to suppress tumor progression via modulation of the immune system, suppression of angiogenesis, induction of apoptosis, and regulation of cellular signaling pathways. Despite the controversy on the role of MSCs in tumor promotion or inhibition, it is obvious that they have a dynamic role in the TME. Considering the wide range of therapeutic applications of MSCs, numerous studies have tried to identify the anti-cancer properties of different types of MSCs. A great body of evidence shows that cancer-naïve MSCs and UC-MSCs have remarkably higher onco-suppressive effects compared with other subtypes. Additionally, some researchers have taken a step further and proposed techniques to convert the tumorigenic function of MSCs into onco-suppressive effects. The existing methods include the application of GM-MSCs, which can help to transfer anti-cancer agents in a highly effective way compared with systemic administration, using MSC-EVs as biocompatible tools to deliver mRNA, miRNAs, prodrugs, and peptides to the target cells, autologous injection of MSCs, which can be administered in combination with prodrugs, therapeutic blockade of cell signaling, and the use of herbal such as curcumin and ginseng. Further studies are suggested to explain the complex interaction between MSCs and tumor components more precisely. Since different subpopulations of MSCs show varied effects, further research should be conducted to evaluate the role of these subpopulations in the progression of different types of cancer which can help to develop more effective methods to convert these unfavorable activities to onco-suppressive effects.
